# Dynamic adaptation of attentional control across bilingual interactional contexts

**DOI:** 10.3389/fpsyg.2026.1860169

**Published:** 2026-10-15

**Authors:** Min Li, Xiangyu Shi, Qinghong Xu, Jie Li

**Affiliations:** 1 School of Psychology, Inner Mongolia Normal University, Hohhot, China; 2 School of Education, Inner Mongolia Minzu University, Tongliao, China; 3 Student Affairs Department, Wuhai Vocational and Technical College, Wuhai, China; 4 School of Foreign Languages, Yulin University, Yulin, China; 5 School of Management, Tianjin University of Traditional Chinese Medicine, Tianjin, China

**Keywords:** adaptation, attentional control, bilingualism, EEG, interactional context

## Abstract

**Background/objectives:**

Bilingual experience is thought to shape cognitive control via adaptive attentional reorganization, yet the neural mechanisms by which different interactional contexts dynamically modulate attentional control remain unclear. This study used EEG to examine how Chinese monolingual, English monolingual, bilingual, and voluntary naming contexts influence attentional control in Chinese–English bilinguals.

**Methods:**

Thirty-five bilinguals completed picture-naming tasks in different contexts, followed by an antisaccade task with concurrent EEG. We analyzed reaction time, ERPs, frontal and parietal delta/theta power, and frontoparietal functional connectivity (wPLI).

**Results:**

Bilingual and voluntary naming contexts shortened antisaccade reaction times (all *p* < 0.01). No significant main effect of context was found on N2 amplitudes (*p* = 0.615). Both contexts reduced parietal delta and theta power relative to the English monolingual context (all *p* < 0.05). Exploratory connectivity analysis initially suggested bilingual-context effects, but this finding did not survive homologous ROI validation. These preliminary sensor-level findings await validation by source-localized EEG or fMRI.

**Conclusions:**

These findings suggest that bilingual and voluntary naming contexts may facilitate attentional control through adaptive reorganization of the attentional system rather than through enhancing a specific inhibitory process. This pattern is consistent with the adaptationist perspective on bilingual cognition and provides preliminary neural evidence for the language mode theory.

## 1 Introduction

Bilingual experience may shape cognitive control, but evidence for a “bilingual advantage” is inconsistent; (Barker and Bialystok, 2019; Donnelly et al., 2019; Lehtonen et al., 2018). If an advantage exists, it may be limited to specific conditions (Paap et al., 2015). One reason is that bilingual experience is heterogeneous. It varies with proficiency, language use, and interactional context (Beatty-Martínez et al., 2020b). Research has therefore shifted from whether an advantage exists to when bilingual experience leads to cognitive change.

Traditionally, bilingual effects have been explained by inhibitory control (Cespón and Carreiras, 2020). Recent accounts propose that bilingual experience may adaptively modulate the attentional system itself (Bialystok, 2024). Frequent engagement of selective attention may improve attentional efficiency, allowing similar or better performance with fewer resources. Attentional control is therefore a core candidate mechanism (Bialystok, 2024). The present study examines how different interactional contexts modulate attentional control and its neural underpinnings.

### 1.1 From bilingual advantage to adaptive control

Early accounts proposed that bilinguals monitor two languages, select the target language, and suppress the non-target language (Green, 1998; Kroll and de Groot, 2005). These demands may strengthen executive function, which led to the bilingual advantage hypothesis (Bialystok, 2017).

However, this hypothesis has faced challenges. Meta-analyses and empirical studies have not consistently replicated bilingual advantages in executive function tasks (Donnelly et al., 2019; Paap et al., 2019). Treating bilingualism as a binary category may overlook its heterogeneity. Factors such as age of acquisition, proficiency, language use, and interactional context may influence cognitive control outcomes (Sulpizio et al., 2020; DeLuca et al., 2020; Hartanto and Yang, 2020).

Research has therefore shifted toward within-group variability and boundary conditions (Grundy, 2020). This shift is consistent with methodological critiques of monolingual-bilingual comparisons (Rothman et al., 2023; Privitera et al., 2026). Such comparisons may make it difficult to identify genuine bilingual effects (Privitera, 2025). The present study follows this approach. It examines within-group variability in attentional control across language modes, rather than comparing bilinguals with monolinguals.

Green and Abutalebi (2013) proposed the adaptive control hypothesis. This hypothesis suggests that different interactional contexts impose different demands on cognitive control (Green and Abutalebi, 2013). These demands may shape the functional architecture of the control system. The research focus has thus moved from how bilingual brains differ to how interactional contexts recruit and shape cognitive control.

### 1.2 Adaptive control hypothesis and contextual effects

The adaptive control hypothesis distinguishes three interactional contexts: single-language, dual-language, and dense code-switching (Green and Abutalebi, 2013). Each context places different demands on cognitive control. In a single-language context, the two languages are used in separate settings. This context mainly requires goal maintenance and interference suppression. In a dual-language context, both languages are used in the same situation, often with different interlocutors. This context requires goal maintenance, interference suppression, and salient cue detection. In a dense code-switching context, speakers mix languages within a single utterance. This context places high demands on opportunistic planning and fine temporal control, rather than on interference suppression.

Some evidence supports this context-specific modulation (Han et al., 2023; Hartanto and Yang, 2020). The ERP study by Jiao et al. is most relevant to the present study. They used a cross-task adaptation paradigm. A picture naming task was interleaved with a flanker task. Compared with single-language contexts, the mixed-language context elicited larger N2 amplitudes and smaller P3/LPC amplitudes in the flanker task (Jiao et al., 2020b). This pattern suggests that language production contexts may modulate executive control. They may enhance earlier cognitive processing and reduce the need for later controlled processing. Together, these findings suggest that different interactional contexts may modulate distinct components of executive function (Jiao et al., 2020a).

However, the adaptive control hypothesis focuses on sustained exposure (Green and Abutalebi, 2013). It does not directly address rapid shifts between language modes. Grosjean's (2023) language mode theory addresses this shorter timescale. The theory proposes that bilinguals adjust language activation along a continuum from monolingual mode to bilingual mode. In monolingual mode, the non-target language is suppressed. In bilingual mode, both languages are active and compete for selection. These shifts can occur quickly and may be non-conscious. Language mode may also affect cognitive processing. Bilingual mode may place greater demands on selective attention than monolingual mode (Cheng and Howard, 2008). In the present study, picture naming was used to place participants in four temporary language modes: Chinese monolingual, English monolingual, bilingual, and voluntary naming. We then examined immediate priming effects on a subsequent attentional control task.

### 1.3 From inhibitory control to attentional control

The present study focuses on attentional control. Early theories, however, explained bilingual effects mainly through inhibitory control. They proposed that bilinguals suppress the non-target language, and that this suppression may strengthen domain-general inhibition (Green, 1998). However, this account has difficulty explaining why bilinguals sometimes perform better on both congruent and incongruent trials (Hilchey and Klein, 2011). Meta-analytic evidence also suggests that bilingual effects extend beyond inhibition to other executive functions, such as working memory and cognitive monitoring (Comishen and Bialystok, 2021; Costa et al., 2009).

Contemporary views therefore place attentional control at the center of bilingual cognitive differences. Attentional control refers to the flexible allocation of cognitive resources, maintenance of goals, and filtering of distraction (Draheim et al., 2022). This view does not reject inhibitory control. Instead, it treats inhibition as one component alongside goal maintenance, monitoring, and resource allocation. In this framework, bilingual effects may reflect a more adaptive optimization of attentional resources, rather than only enhanced interference suppression (Bialystok and Craik, 2022).

Bilingual experience may also optimize the attentional system as a whole, rather than modify a single control module (Bialystok, 2024). Inhibitory control can be seen as one component of this broader system. The modified attentional monitoring model (Stuss et al., 1995) offers a useful description. The monitoring system is engaged when people need to select among schemas or suppress inappropriate ones. It involves schema activation, inhibition, and feedback-based regulation. From this perspective, attentional control involves sustained top-down monitoring. This monitoring may be particularly important when tasks require decision-making, planning, error correction, or overcoming automatic responses. In bilingual contexts, selective attention is engaged frequently. This engagement may help the monitoring system optimize its efficiency (Bialystok, 2024). Thus, bilingual experience may optimize the operating mode of the attentional control system, rather than train a specific ability.

This dynamic view is further developed by the malleable attentional resources theory (Young and Stanton, 2002). The theory suggests that attentional resources are not fixed. They can be regulated according to task demands. When cognitive load is below a person's regulatory capacity, resources may be consumed passively. This can lead to temporary declines in performance. When cumulative load exceeds a critical threshold, the system may reorganize and expand its resources. This reorganization may form a more efficient control mode that carries over to later tasks. Applied to bilingualism, this threshold principle suggests that brief exposure to high linguistic load may not be enough to trigger adaptation. More sustained high load may be needed for stable cognitive gains.

At the neural level, attentional control is supported by the frontoparietal network (FPN). This network includes lateral pre-frontal and posterior parietal regions (Corbetta and Shulman, 2002; Scolari et al., 2015). The two hemispheres may contribute differently. The right hemisphere is involved in attention to both hemifields, whereas the left hemisphere mainly directs attention to the right (Heilman and Van Den Abell, 1979). The dual-network model distinguishes a bilateral dorsal attention network (DAN) for goal-directed attention and a right-lateralized ventral attention network (VAN) for reorienting to salient stimuli (Corbetta and Shulman, 2002, 2011). The balance between hemispheres is not fixed. It can be reconfigured by internal states (Niedernhuber et al., 2025). This framework motivates the use of functional connectivity to examine whether interactional contexts reshape left-right balance in FPN engagement. The dual-network model suggests that if right-hemispheric VAN resources are challenged, interhemispheric balance within the DAN may shift, which may appear as left-right differences in connectivity.

### 1.4 The antisaccade task as a paradigm for measuring attentional control

The antisaccade task is widely used to measure attentional control (Hutton, 2008). Participants are asked to suppress a reflexive saccade toward a peripheral stimulus and instead make a voluntary saccade in the opposite direction (Hallett, 1978). Performance depends on inhibiting a prepotent response and executing a goal-directed action (Draheim et al., 2021). The task requires maintaining task goals and suppressing interference (Kane et al., 2001). It has good internal consistency and test-retest reliability. It also loads on a latent attentional control factor (Draheim et al., 2021). In bilingualism research, the task has been used to examine attentional control (Bialystok and Craik, 2022; Cook et al., 2025).

EEG can capture the neural dynamics of attentional control during the antisaccade task. When averaged to stimulus onset, the task elicits a sustained negativity over central and frontal electrodes. This negativity is larger for antisaccades than for prosaccades (Papadopoulou et al., 2010). It may vary with response preparation. Faster antisaccades have been associated with larger negativity. This negativity may reflect cortical control processes involved in response inhibition and goal maintenance. Its time window and scalp distribution overlap with the N2 component. The N2 is often linked to conflict monitoring and inhibitory control (Donkers and Van Boxtel, 2004; Folstein and Van Petten, 2008). Given this overlap, we quantified the mean amplitude of this fronto-central negativity over the N2 time window. We refer to it as the N2-like negativity. This measure can index preparatory attentional control processes in the present study.

Beyond ERPs, theta and delta oscillations provide additional information about attentional control. Midfrontal theta (4–8 Hz) has been linked to cognitive control, conflict monitoring, and response inhibition (Cavanagh and Frank, 2014). It may also reflect the allocation of control resources. Parietal theta has been associated with spatial attention and the suppression of irrelevant information (Asanowicz et al., 2023; Haciahmet et al., 2021). In the antisaccade task, parietal theta may index attentional resource allocation for spatial processing and target selection. Delta oscillations (2–4 Hz) have also been linked to attentional and inhibitory processes (Harmony, 2013). Their role may depend on state. Moderate delta may help exclude distraction, whereas excessive delta may reflect lower alertness (Luna et al., 2023). The present study therefore examined whether language contexts modulate attentional control through delta oscillations.

Attentional control also depends on coordinated activity across brain regions, especially the frontoparietal network (Corbetta and Shulman, 2002). We used the weighted phase lag index (wPLI) to measure functional connectivity. wPLI quantifies phase synchronization between brain regions and is robust to volume conduction (Vinck et al., 2011). According to the communication-through-coherence hypothesis, phase synchronization may support selective communication between regions (Fries, 2015). In attentional control tasks, frontoparietal connectivity may reflect the coordination of top-down control (Carter et al., 2023). In the antisaccade task, frontal and parietal regions are involved in response suppression and goal maintenance (Abutalebi and Green, 2007). Thus, wPLI in these regions can serve as an exploratory measure of network-level efficiency.

### 1.5 The present study

This study examined whether different bilingual interactional contexts may trigger adaptation of the attentional system, and which neural mechanisms may be involved. We considered three levels of analysis: behavioral performance, oscillatory dynamics, and exploratory connectivity. We used a within-subjects design. Participants performed picture naming in four contexts: Chinese monolingual, English monolingual, bilingual cued switching, and voluntary naming. Each context was followed by an antisaccade task with concurrent EEG.

Based on the adaptive control hypothesis (Green and Abutalebi, 2013), we designed four interactional contexts. Cue-prompted naming was used to create monolingual and bilingual contexts (Jiao et al., 2020b). Voluntary naming was used to create a context similar to dense code-switching (Beatty-Martínez et al., 2020a; De Bruin et al., 2018). The four contexts differ in language control demands. In the monolingual contexts, participants named pictures in one language and suppressed the other. In the bilingual context, participants switched between languages according to color cues. This context required goal maintenance, interference suppression, and cue detection. In the voluntary naming context, participants chose the language freely. This context involved dual-language activation but no external switching demands (Kennis et al., 2025). This design allowed us to examine whether attentional modulation varies with language control demands.

The four dependent measures capture attentional modulation at different levels: behavioral efficiency, early conflict monitoring, sustained oscillatory dynamics, and exploratory connectivity. Reaction times provide a general index of attentional efficiency. The N2 component may index early conflict monitoring (Donkers and Van Boxtel, 2004; Folstein and Van Petten, 2008). Theta and delta power may reflect sustained resource allocation and inhibitory processing (Cavanagh and Frank, 2014; Harmony, 2013). Frontoparietal wPLI was used as an exploratory measure of sensor-level connectivity (Vinck et al., 2011). Together, these measures cover different spatiotemporal scales, from transient responses to sustained oscillations and exploratory connectivity.

We addressed three comparisons, each linked to a hypothesis. First, we compared bilingual with monolingual contexts to test whether cued switching creates enough control load to exceed the reorganization threshold. We expected that bilingual and voluntary naming contexts would be associated with faster antisaccade reaction times than monolingual contexts (Young and Stanton, 2002). Second, we compared voluntary naming with monolingual contexts to test whether dual-language co-activation alone is sufficient to trigger adaptation. At the electrophysiological level, we examined the N2 component as an index of early conflict monitoring. Previous studies suggest that ERP effects may be less sensitive to within-group differences (Pereira Soares et al., 2024; Xie et al., 2024). We therefore also examined theta and delta power. Reduced theta and delta power may be a more sensitive indicator of neural efficiency (Cohen, 2011; Gratton et al., 2018). Third, we compared bilingual with voluntary naming contexts to test whether facilitation varies with control intensity. Given the dual-network model (Corbetta and Shulman, 2002, 2011), we explored whether interactional contexts modulate left-right frontoparietal connectivity. Because EEG connectivity is sensor-level and no prior ROI hypotheses were available, this analysis was exploratory. The two monolingual contexts were not compared with each other, as both served as baselines.

By integrating these behavioral and neural measures across four interactional contexts, the present study aims to provide a more complete account of how—and at which levels—bilingual language contexts dynamically reshape attentional control, offering empirical evidence for the dynamic adaptation of the bilingual brain across interactional contexts.

## 2 Materials and methods

### 2.1 Participants

A priori sample size estimation was conducted using G^*^Power 3.1 (Faul et al., 2007) for a one-way repeated measures ANOVA with four within-subject conditions (Chinese monolingual, English monolingual, bilingual, and voluntary naming contexts). Based on an assumed medium effect size (*f* = 0.25), α = 0.05, power (1-β) = 0.80, and the default correlation among repeated measures (*r* = 0.5), the analysis indicated a required sample size of 24 participants. Forty independent bilingual Chinese-English speakers from Inner Mongolia University of Nationalities were selected for the experiment (14 males, 26 females). All participants were right-handed with normal or corrected vision. Their native language was Mandarin, and they had all passed the College English Test Band 4 (CET-4), a nationally standardized English proficiency test administered by the Ministry of Education in China, but had not passed CET-6. The CET-4 requires a vocabulary of approximately 4,200 words and tests listening, reading, writing, and translation skills, with a pass threshold of 425 out of 710 points. By selecting participants who passed CET-4 but not CET-6, we ensured a relatively homogeneous sample with intermediate English proficiency.

Language proficiency was assessed using a 10-point self-rating scale (1 = very unskilled, 10 = very skilled). Participants rated their Mandarin (L1) and English (L2) abilities across four dimensions: listening, speaking, reading, and writing. The overall proficiency score for each language was calculated as the mean of these four ratings.

Socioeconomic status (SES) was measured using both subjective and objective indices. Subjective SES was rated on a 10-point scale (1 = lowest, 10 = highest), where participants compared their family's social standing to others in Chinese society. Objective SES was assessed based on parental education and occupation, following the procedure described by Shi and Shen (2007). Parental education was coded on a 6-point scale (1 = no formal education, 2 = elementary school, 3 = middle school, 4 = high school or secondary technical school, 5 = college or university, and 6 = graduate degree or above). Parental occupation was coded on a 5-point scale (1 = temporary worker, 2 = self-employed, 3 = general professional or technical staff, 4 = mid-level professional or technical staff, and 5 = senior professional or technical staff). The total objective SES score was calculated as the sum of these four components (father's education + mother's education + father's occupation + mother's occupation), yielding a possible range from 4 to 22 (education: 2–12, occupation: 2–10), with higher scores indicating higher socioeconomic status. Additional demographic measures included participants' age, age of English acquisition, and Raven's Standard Progressive Matrices scores, which are summarized in Table 1.

**Table 1:** Demographic and language background characteristics of participants (*N* = 40).

Subject information	*M*	SD	min	max
Age	19.90	1.41	17	22
Age of English acquisition	8.31	2.45	6	12
Self-rated Chinese proficiency	8.75	1.45	4	10
Self-rated English proficiency	6.06	1.73	2.5	9
Objective SES	10.67	3.44	5	20
Subjective SES^*^	5.00	1.46	2	8
Raven's test	12.85	3.8	8	16

^*^Subjective SES was rated on a scale of 1 to 10 (1 = lowest, 10 = highest). Objective SES ranged from 4 to 22 (based on parental education and occupation). Raven's test consisted of 18 odd-numbered items from Sets C, D, and E, with a maximum score of 18.

All participants signed informed consent forms. The study was approved by the Academic Committee of the School of Education Sciences at Inner Mongolia Minzu University (Approval No. NMDJKY-RT-2022-10-11).

### 2.2 Design

The experiment employed a single-factor within-subjects design. The independent variable, interactional context, was manipulated across four levels: Chinese monolingual context, English monolingual context, bilingual context, and voluntary naming context. The primary dependent variables were reaction time and accuracy on the antisaccade task, as well as EEG measures including event-related potentials (N2 component), time-frequency power (delta and theta bands), and frontoparietal functional connectivity (wPLI).

We employed cue-prompted naming tasks to induce monolingual and bilingual contexts. Monolingual contexts comprised Mandarin-only picture naming and English-only picture naming, in which the bilingual participants performed monolingual naming based on color cues. Bilingual contexts involved naming in the corresponding language based on different color cues (Jiao et al., 2020b). In the voluntary naming context, participants were instructed to freely choose between Mandarin and English for each picture, following voluntary switching paradigms (De Bruin et al., 2018). Unlike the bilingual context (cued switching), this condition involved free choice rather than forced switching across multiple trials. Note that switch rates were not calculated or analyzed, as the primary goal was to prime a voluntary language choice context rather than to quantify switching behavior *per se*.

### 2.3 Materials and procedure

The experimental materials consisted of 88 black-and-white line drawings (Zhang and Yang, 2003). The images depicted concepts from common semantic categories, including 11 categories: “fruits, tools, clothing, furniture, transportation, poultry, vegetables, toys, body organs, stationery, and mammals.”

Different interactional contexts were primed using picture naming tasks. After completing picture naming (language production) tasks, participants performed a anti-saccade task. Prior to the experiment, participants were thoroughly familiarized with the Mandarin and English naming conventions for the pictures. Reaction times and accuracy rates for both Mandarin and English picture naming were checked to ensure participants were familiar with the experimental materials. Participants were sequentially presented with 88 pictures and their corresponding Chinese (or English) labels. After one full round of presentation, the same pictures and labels were presented again in sequence. Following the learning phase, participants were shown only the pictures and asked to report both the Chinese and English names of the pictures. If a participant's accuracy rate for either Chinese or English naming fell below 90%, they were required to re-familiarize themselves with the stimulus materials until their accuracy rate reached 90% or higher. Subsequently, participants completed practice sessions that resemble the formal experiment, consisting of 32 trials and using 8 different images as stimuli. The sequence of Chinese and English presentations remains consistent across the familiarization, practice, and formal experiment phases and is balanced across participants.

The experimental procedure consisted of four blocks, corresponding to four priming conditions: Chinese monolingual context, English monolingual context, bilingual context, and voluntary naming context. To control for potential order effects while preventing carryover effects from the switching contexts contaminating the monolingual baseline, the presentation order of the four blocks was arranged using a Latin Square design. This design ensured that each of the four context conditions appeared equally often in each ordinal position (first, second, third, and fourth) across participants, while also ensuring that each condition preceded and followed every other condition an equal number of times. The Latin Square yielded four distinct presentation sequences, which were randomly assigned to participants. This counterbalancing approach allowed for the systematic examination of context effects while minimizing the influence of fatigue, practice, or carryover effects on attentional control performance.

Within each block, each trial cycle consisted of two consecutive picture naming trials followed by two consecutive anti-saccade trials. Each block contained 60 such cycles, resulting in 120 picture naming trials and 120 anti-saccade trials per block. The antisaccade task was embedded within each language context to measure attentional control efficiency immediately following the priming of different interactional contexts, allowing us to assess context-dependent carryover effects on subsequent executive control performance. In the picture naming task, two consecutive images appeared sequentially at the center of the screen. Participants named the image in Chinese or English based on the color of the prompt box. In the Chinese monolingual context: all trials presented a red border, and participants named the images in Chinese. In the English monolingual context: all trials presented a blue border, and participants named the images in English. In the Bilingual context: red and blue borders appeared alternately and randomly across trials, and participants named the images in the language indicated by the border color. In the voluntary naming context: red and blue half-frames appeared simultaneously on the screen, with red and blue alternating between top and bottom positions across trials. Participants were free to choose to name each image in either Chinese or English. In the anti-saccade task, participants were instructed to ignore the actual location of a star symbol and instead look toward the opposite position.

Each trial commenced with a 300 ms fixation point at the screen center, followed by a 100 ms blank screen, and then an 1,800 ms image stimulus for the picture naming task. Participants were required to verbally identify the image they observed. Following the picture naming trial, a 100 ms blank screen appeared, followed by a fixation point presented for a random duration between 600 and 1,000 ms. A 300 ms asterisk then appeared at a 12° visual angle to the left or right of the fixation point. Immediately after, the target letter “Q” or “O” appeared for 100 ms on the opposite side of the asterisk. A “#” symbol then masked the positions of both the asterisk and target letter for 300 ms. When “&&&&” appeared on the screen, the participant was required to respond with a keypress within 1500 ms. After a 1000 ms blank screen, the next trial began. The participant's task was to ignore the asterisk and shift their gaze to the opposite side of the screen to search for the target letter “Q” or “O”. If the target was “Q”, they pressed the “Q” key; if the target was “O”, they pressed the “O” key (Figure 1).

**Figure 1:**
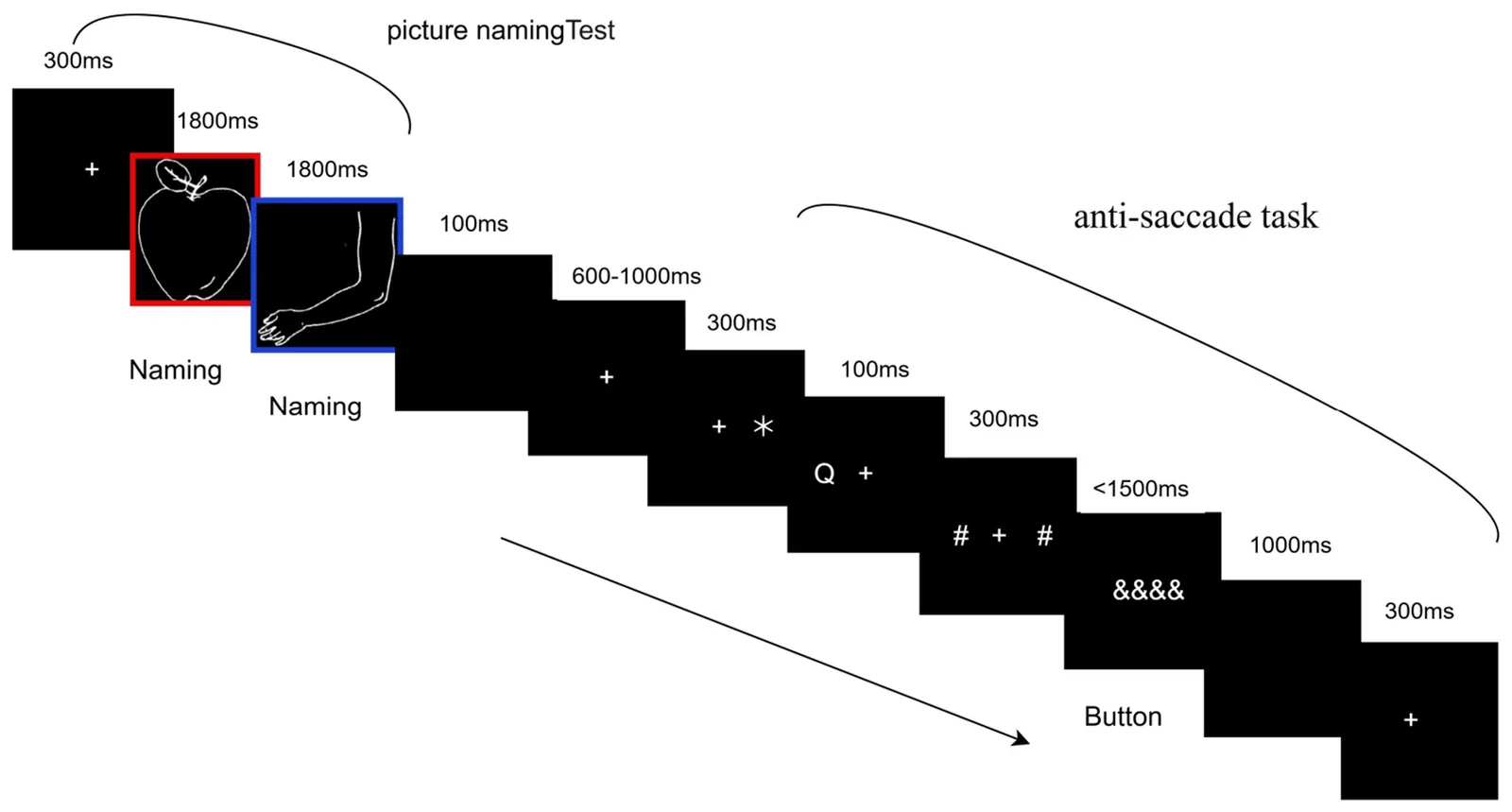
Experiment flowchart.

### 2.4 EEG acquisition and preprocessing

EEG signals were recorded using a Neuroscan 64-channel acquisition system with a 64-electrode cap arranged according to the extended international 10–20 system. The sampling rate was 1,000 Hz, with an online bandpass filter of 0.05–100 Hz. Online recordings were referenced to the global average. Vertical and horizontal electrooculograms (VEOG and HEOG) were simultaneously recorded. Electrode impedances were maintained below 5 kΩ.

Data were preprocessed offline using the EEGLAB toolbox (Delorme and Makeig, 2004). The data were downsampled to 500 Hz and re-referenced to the bilateral mastoids (M1, M2). A zero-phase low-pass filter at 30 Hz was applied. Ocular and muscular artifacts were removed using independent component analysis (ICA). Time-frequency analysis was performed using Morlet wavelet transform (Cohen, 2014) to obtain dynamic changes in EEG power in the time-frequency domain.

### 2.5 Statistical analyses

Statistical analyses were performed using JASP 0.18.3.0 software. One-way repeated measures ANOVAs with context as the within-subjects factor were conducted on behavioral data (Jiao et al., 2020a,b), time-frequency power (Cohen, 2014), and functional connectivity strength (Vinck et al., 2011). When ANOVA results were significant, paired-sample *t*-tests were used for *post-hoc* comparisons with Bonferroni correction for multiple comparisons. Effect sizes are reported as Cohen's d and η^2^p.

Time-frequency analysis was performed using Morlet wavelet transform (Cohen, 2014) to obtain dynamic changes in EEG power in the time-frequency domain. Epochs were segmented from −1500 ms to +2000 ms relative to stimulus onset. The frequency range was set from 2 to 30 Hz in 1 Hz steps. To achieve an optimal balance between time and frequency resolution across frequency bands, the number of wavelet cycles was dynamically adjusted, linearly increasing from 1.5 cycles at 2 Hz to 7 cycles at 30 Hz. Baseline correction was performed using the pre-stimulus window from −500 ms to −200 ms.

Frontal and parietal electrode selection for time-frequency analysis was guided by their distinct functional contributions to cognitive control. Frontal regions, particularly the anterior cingulate and dorsolateral prefrontal cortices, are critically involved in conflict monitoring and the implementation of cognitive control, processes that are reflected in midline frontal theta oscillations (Carter and Van Veen, 2007; Cavanagh and Frank, 2014). Parietal regions, by contrast, play a central role in spatial attention and the integration of sensory information, with parietal theta and delta oscillations reflecting attentional resource allocation and the suppression of task-irrelevant information (Haciahmet et al., 2021; Yang et al., 2024). Accordingly, frontal electrodes (Fz, FCz, F1, F2, F3, F4) and parietal electrodes (Pz, CPz, P1, P2, P3, P4) were selected based on the international 10–20 system to provide representative sampling of these functionally distinct regions. The time window of 300–600 ms post-stimulus was chosen to capture the full time course of attentional monitoring processes following the onset of the asterisk. Mean power in the delta (2–4 Hz) and theta (4–8 Hz) frequency bands was extracted from this 300–600 ms time window for statistical analysis.

The present study employed the weighted phase lag index (wPLI) to calculate functional connectivity between brain regions (Vinck et al., 2011). The traditional phase lag index (PLI) quantifies functional connectivity by evaluating the asymmetry of the phase difference distribution between two signals but is sensitive to minor phase noise. wPLI improves upon this by weighting the imaginary part of the cross-spectrum, not only inheriting PLI's advantage of effectively eliminating volume conduction effects but also further reducing artifacts caused by unrelated noise, thus more accurately reflecting true neural synchronization.

Specifically, for any two EEG channels x(t) and y(t), their cross-spectrum X_xy_=Z_x_${\text{Z}}_{\text{y}}^{*}$ in the frequency domain is first calculated, where Z is the complex signal obtained through time-frequency transformation, and ^*^ denotes the complex conjugate. wPLI is defined as the ratio of the absolute value of the mean imaginary part of the cross-spectrum to the mean of the absolute values of the imaginary part of the cross-spectrum, as shown in Equation 1:


$$\begin{array}{c} wPLI=\frac{\left|E\left\{Im\left(X_{xy}\right)\right\}\right|}{E\left\{\left|Im\left(X_{xy}\right)\right|\right\}} \end{array}$$ (1)

Here, Im(X_xy_) denotes the imaginary part of the cross-spectrum, and E{•} represents the expectation across all trials. The value of wPLI ranges from 0 to 1, with higher values indicating greater phase synchronization between two brain regions in that frequency band.

Functional connectivity analysis was conducted in two stages. First, an exploratory data-driven analysis was performed using point-to-point scanning across all frontal-parietal electrode pairs to identify electrode clusters showing the most robust context-dependent differences. Second, to further examine the consistency of the observed patterns and to address potential electrode-number confounds, we repeated the analysis using strictly homologous electrode clusters based on the international 10–20 system.

The electrode selections for different analyses were guided by the specific theoretical questions each analysis was intended to address. For the N2 component, which is theoretically a fronto-central global component without established hemispheric asymmetry (Donkers and Van Boxtel, 2004; Folstein and Van Petten, 2008), we averaged across fronto-central electrodes without differentiating hemispheres. For time-frequency analysis, we distinguished frontal from parietal regions because frontal theta reflects conflict monitoring whereas parietal theta/delta reflects spatial attention and inhibition (Asanowicz et al., 2023; Haciahmet et al., 2021); hemispheric differences were not theoretically predicted for these measures. For functional connectivity analysis, however, we explicitly distinguished left and right hemispheres because this analysis was designed to test the dual-network model (Corbetta and Shulman, 2002, 2011), which makes specific predictions about right-hemispheric ventral attention network function and interhemispheric balance. Thus, the differences in electrode selection across analyses reflect a theory-driven, multi-level analytical strategy rather than arbitrary choices.

## 3 Results

### 3.1 Behavioral results

Reaction time data from the anti-saccade task were analyzed. Trials with incorrect responses, as well as those with reaction times shorter than 100 ms or longer than 1,000 ms, were excluded from analysis. Five participants with more than 25% of trials removed were excluded from further analysis, resulting in a final sample of 35 participants. For each condition, trials with reaction times exceeding ±3 standard deviations from the mean were additionally removed as outliers, leading to the exclusion of approximately 5% of the data.

A one-way repeated measures ANOVA on reaction time data revealed a significant main effect of interactional context, *F*(3, 102) = 9.485, *p* < 0.001, η^2^p = 0.218. *Post-hoc* tests revealed that reaction times in the bilingual context were significantly faster than in the English monolingual context, *t*(34) = 4.236, *p* < 0.001, Cohen's *d* = 0.560; reaction times in the bilingual context were significantly faster than in the Chinese monolingual context, *t*(34) = 4.019, *p* < 0.001, Cohen's *d* = 0.531; reaction times in the voluntary naming context were significantly faster than in the English monolingual context, *t*(34) = 3.432, *p* = 0.003, Cohen's *d* = 0.453; and reaction times in the voluntary naming context were significantly faster than in the Chinese monolingual context, *t*(34) = 3.215, *p* = 0.005, Cohen's *d* = 0.425 (Table 2, Figure 2).

**Table 2:** Anti-saccade reaction times and accuracy rates (M ± SD)under different contextual conditions.

Measure	English monolingual context	Chinese monolingual context	Bilingual context	Voluntary naming context
Reaction time (ms)	280.96 ± 119.81	277.68 ± 131.89	217.01 ± 95.70	229.15 ± 106.34
Accuracy (%)	0.92 ± 0.12	0.91 ± 0.15	0.94 ± 0.13	0.93 ± 0.13

**Figure 2:**
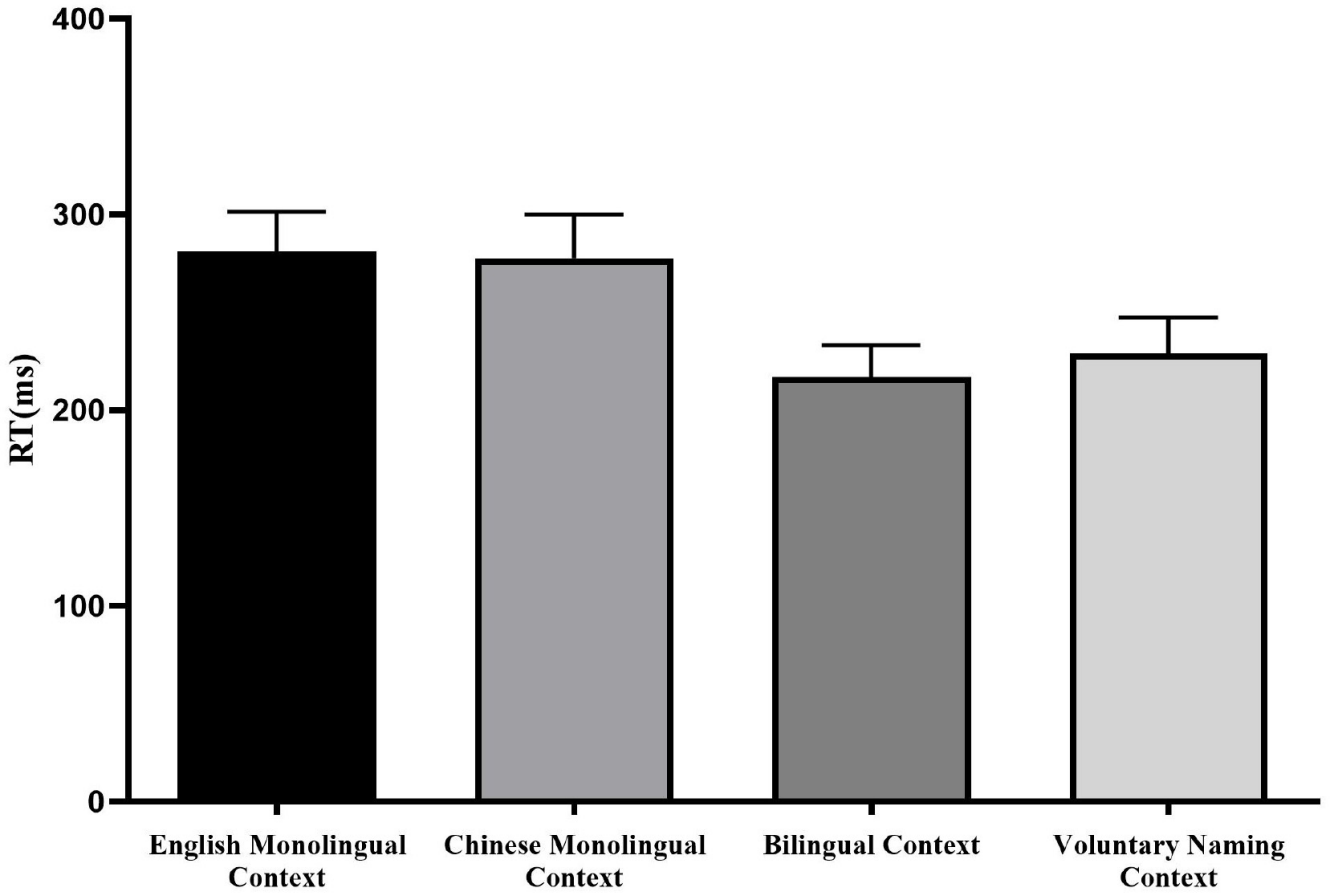
Reaction times in the anti-saccade task under different context conditions.

A one-way repeated measures ANOVA on accuracy data revealed a non-significant main effect of interactional context, *F*(3, 102) = 1.27, *p* = 0.288, η^2^p = 0.036.

### 3.2 Electrophysiological results

#### 3.2.1 Event-related potential results

Based on previous ERP findings, the present study primarily focused on the N2 component. In combination with the grand-average waveform collapsed across all four experimental conditions, the time window for the N2 component was defined as 250–320 ms. The grand-average waveform showed a prominent negative-going deflection beginning at approximately 250 ms. Following the approach described by Luck and Gaspelin (2017), the time window was determined independently of condition-related differences. Guided by the scalp topography within this time window, the mean amplitude of the N2 component was quantified at frontocentral electrodes (Fz, FCz, FC1, and FC2). Figure 3 illustrates the mean amplitudes of the N2 component within the 250–320 ms time window across different contextual conditions in the antisaccade task. Figure 4 illustrates the corresponding scalp topographies within the same time window.

**Figure 3:**
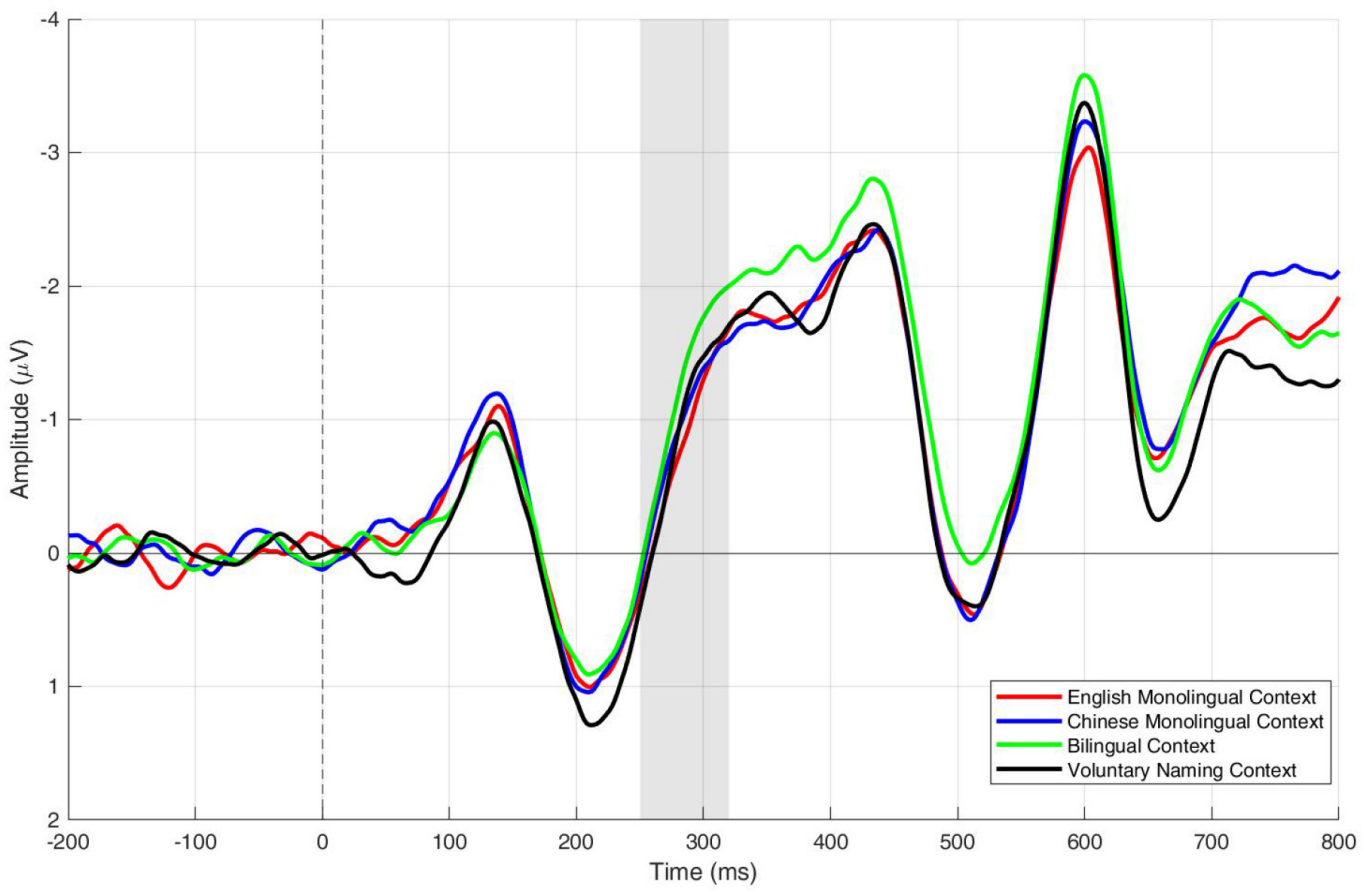
Grand-averaged ERP waveforms at fronto-central electrodes (Fz, FCz, FC1, FC2) during the antisaccade task across the four language contexts.

**Figure 4:**
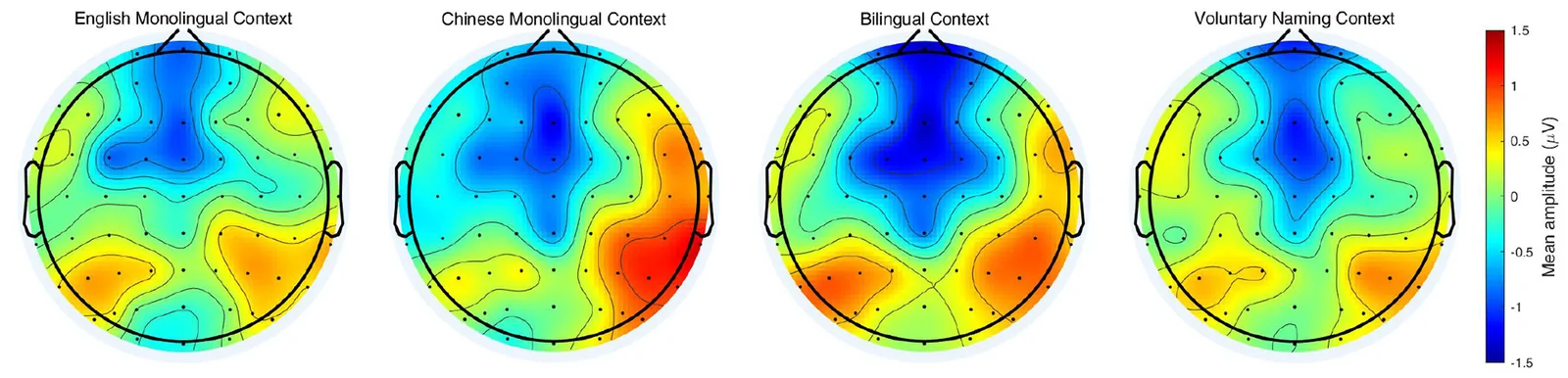
Topographical distribution of N2 amplitude (250–320 ms) across language contexts.

A one-way repeated-measures analysis of variance (ANOVA) was conducted on the mean amplitudes of the N2 component averaged across the fronto-central electrodes (Fz, FCz, FC1, and FC2). The results revealed that the main effect of condition was not statistically significant, *F*(3, 102) = 0.603, *p* = 0.615, $\eta_{\text{p}}^{2}$ = 0.017.

#### 3.2.2 Time-frequency analysis results

Mean delta (2–4 Hz) and theta (4–8 Hz) power was extracted from the frontal and parietal electrodes within the 300–600 ms post-stimulus time window. One-way repeated measures ANOVAs on delta band (2–4 Hz) mean power were conducted separately for frontal and parietal regions. Frontal delta power did not differ significantly across contexts [*F*(3, 102) = 2.132, *p* = 0.101, η^2^p = 0.059].

Delta band activation in the parietal region was relatively stable, with a significant main effect of interactional context [*F*(3, 102) = 7.249, *p* < 0.01, η^2^p = 0.176]. *Post-hoc* tests revealed that the English context elicited the strongest delta (2–4 Hz) oscillation, with power values significantly greater than in the Chinese context [*t*(34) = 2.570, *p* = 0.046, Cohen's *d* = 0.306]; significantly greater than in the bilingual context [*t*(34) = 3.710, *p* = 0.002, Cohen's *d* = 0.441]; and significantly greater than in the voluntary naming context [*t*(34) = 4.299, *p* < 0.001, Cohen's *d* = 0.512](Figure 5).

**Figure 5:**
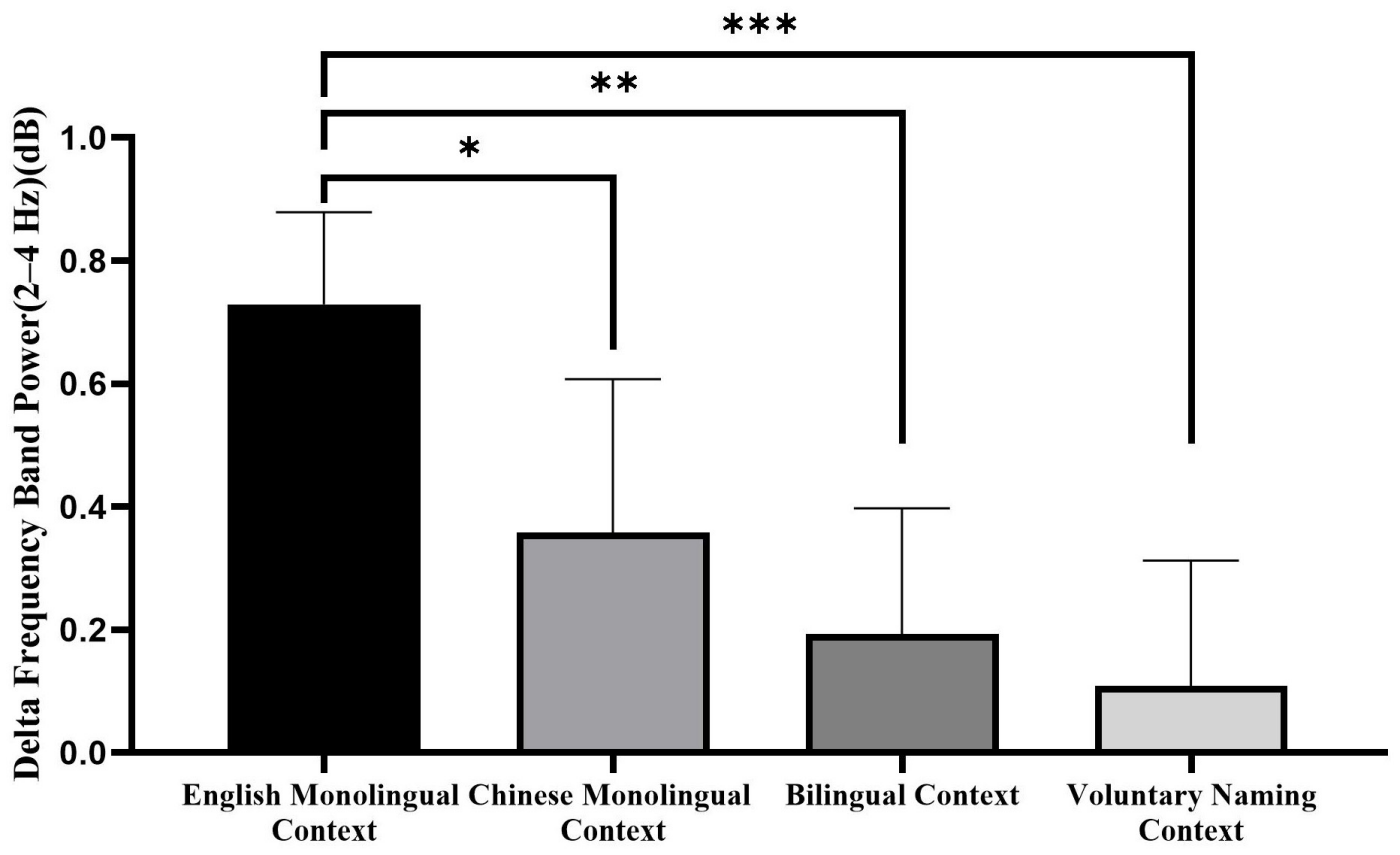
Parietal delta power (2–4 Hz) across the four language contexts. ^*^p < 0.05; ^**^p < 0.01; ^***^p < 0.001.

One-way repeated measures ANOVAs on theta band (4–8 Hz) mean power were conducted separately for frontal and parietal regions. Frontal region analysis revealed a significant main effect of interactional context [*F*(3, 102) = 2.759, *p* = 0.046, η^2^p = 0.075], but *post-hoc* tests did not reveal any significant pairwise comparisons. Given the absence of significant pairwise differences, this effect should be interpreted with caution.

Parietal region analysis revealed a significant main effect of interactional context [*F*(3, 102) = 3.764, *p* = 0.013, η^2^p = 0.100]. *Post-hoc* tests revealed that the English context elicited the strongest theta band neural oscillations, with power values significantly greater than in the bilingual context [*t*(34) = 2.880, *p* = 0.029, Cohen's *d* = 0.318] and marginally significantly greater than in the voluntary naming context [*t*(34) = 2.606, *p* = 0.053, Cohen's *d* = 0.288] (Figure 6).

**Figure 6:**
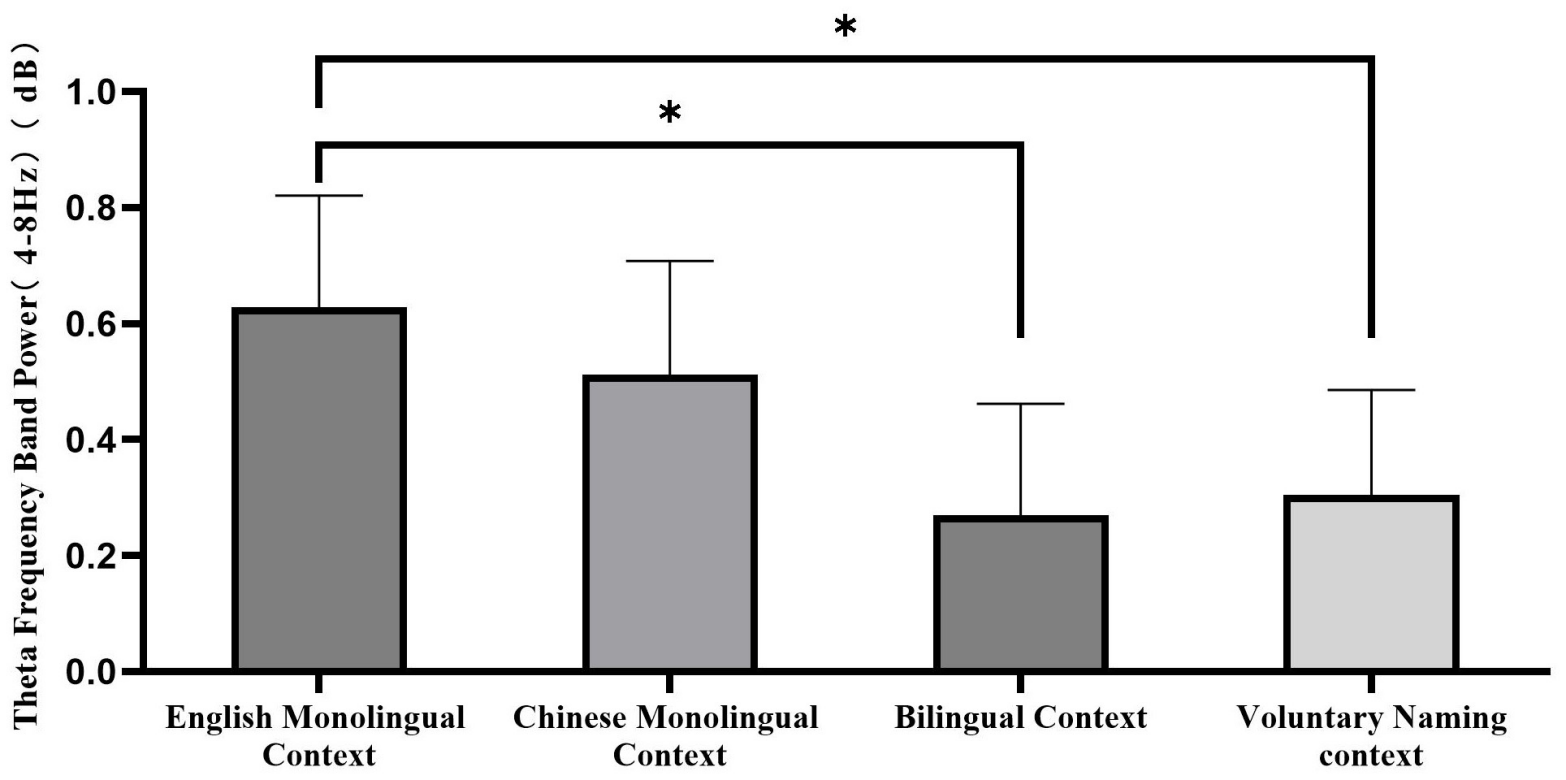
Parietal theta power (4–8 Hz) across the four language contexts. ^*^p < 0.05; ^**^p < 0.01; ^***^p < 0.001.

Figure 7 shows the time-frequency representations of delta and theta power at frontal electrodes across the four language contexts. Figure 8 shows the corresponding time-frequency representations at parietal electrodes. Figure 9 presents the scalp topographies of mean delta and theta power, averaged over the 300–600 ms post-stimulus window and the respective frequency bands, for each language context.These topographic maps complement the time-frequency plots by showing the scalp distribution of oscillatory power for each context. They are based on all electrodes, whereas statistical analyses focused on frontal and parietal regions.

**Figure 7:**
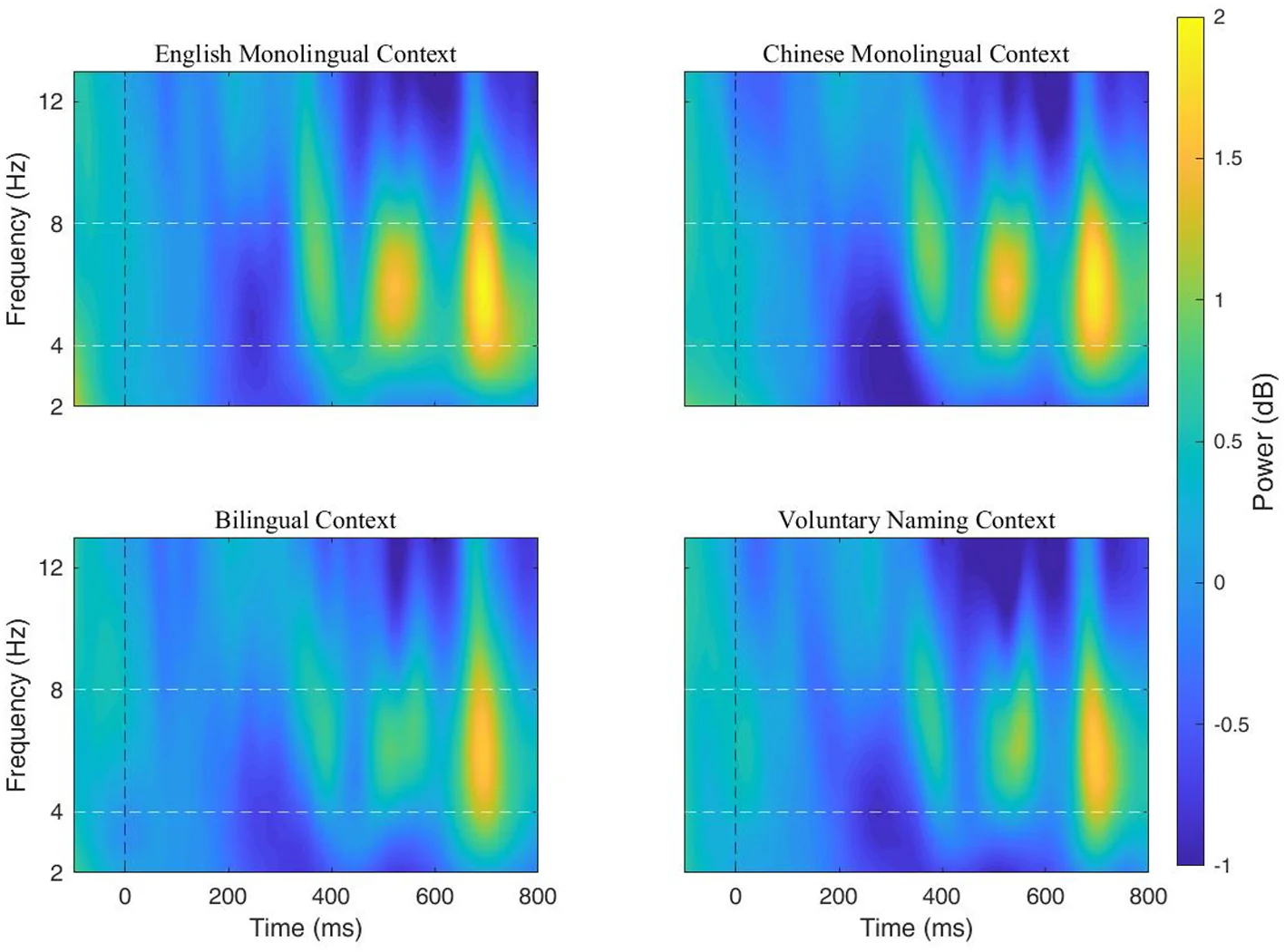
Time-frequency plots of EEG power at frontal electrodes across the four language contexts.

**Figure 8:**
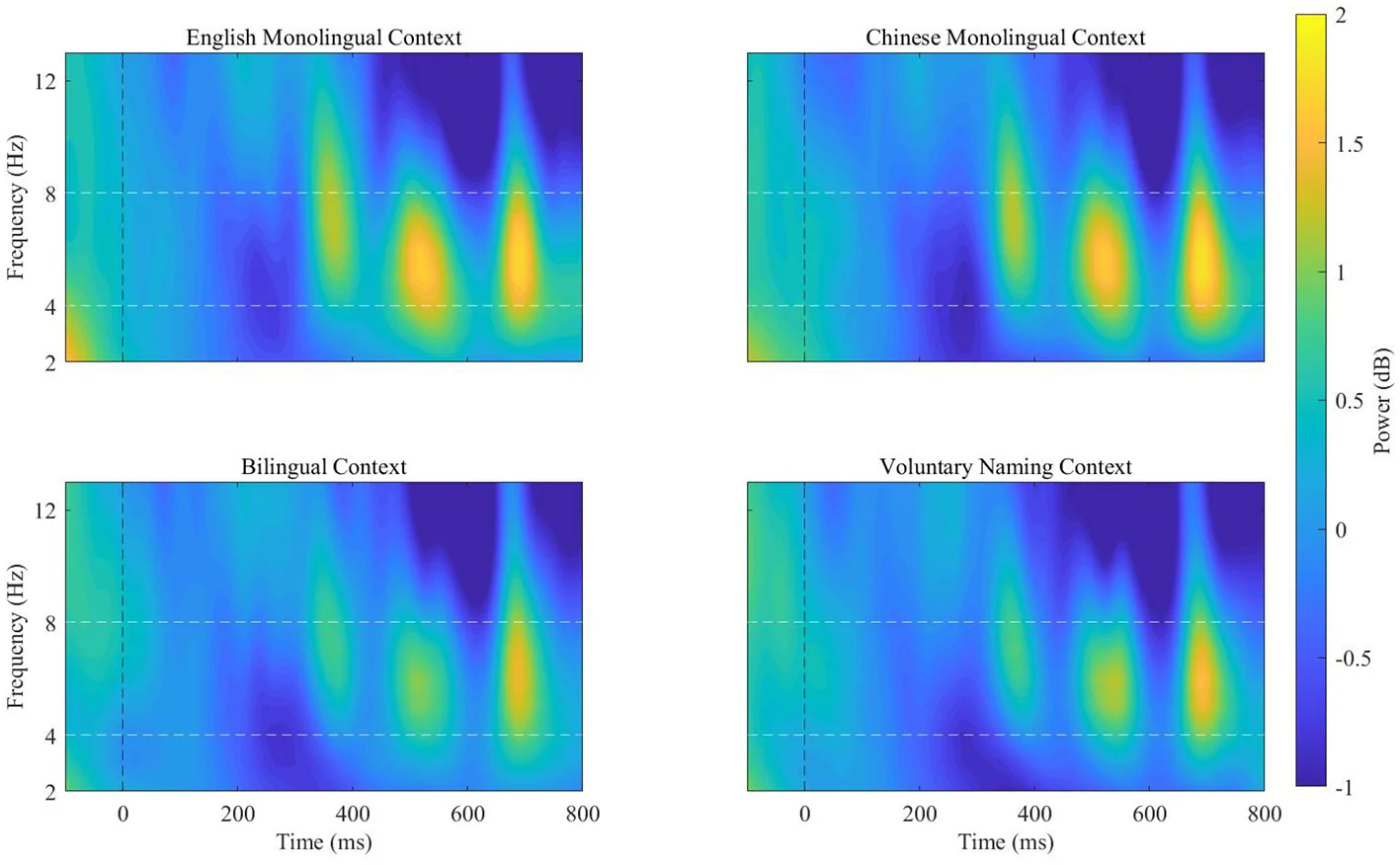
Time-frequency plots of EEG power at parietal electrodes across the four language contexts.

**Figure 9:**
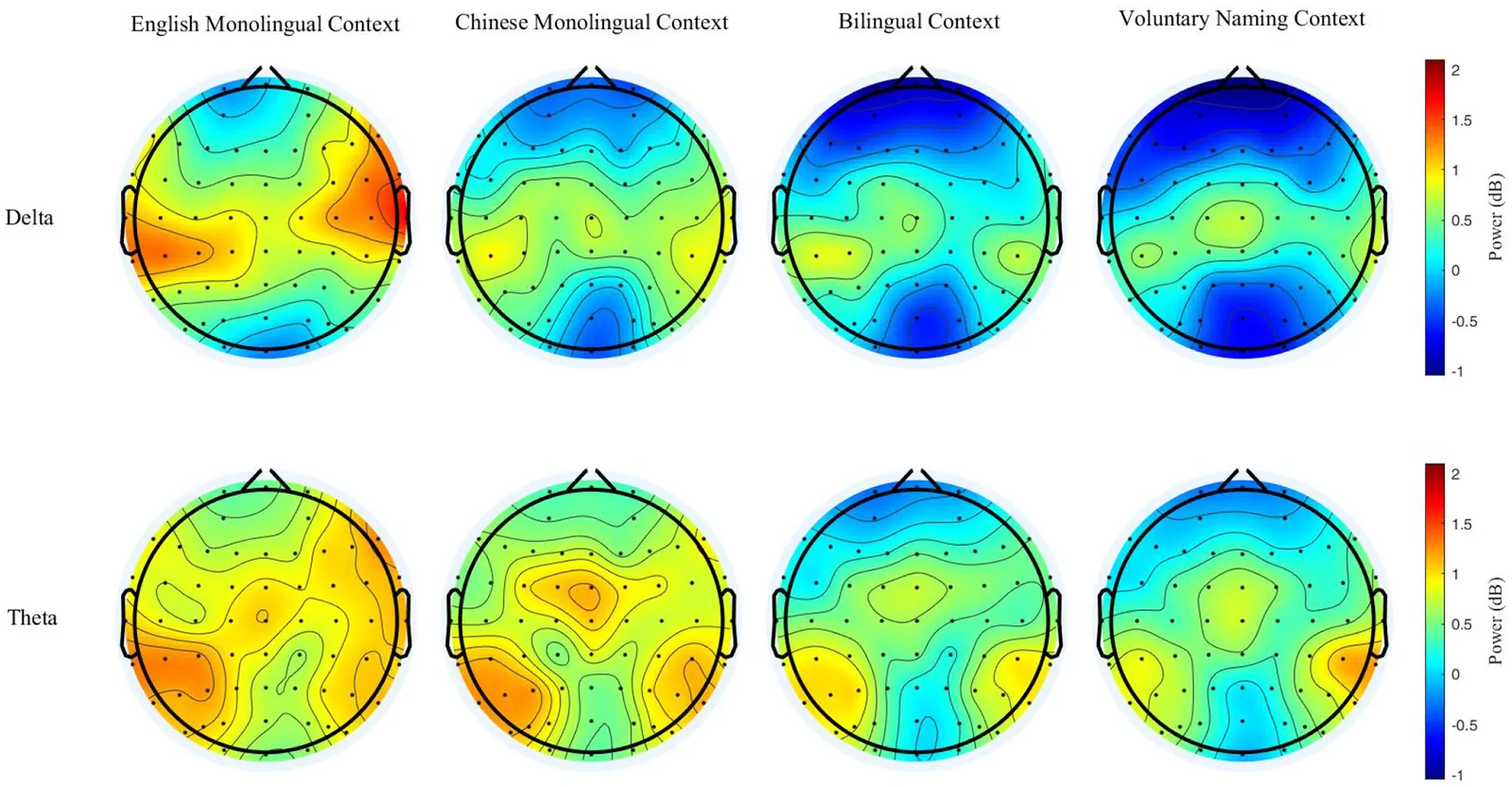
Topographical distribution of mean delta (2–4 Hz, top) and theta (4–8 Hz, bottom) power across the four language contexts (300–600 ms).

#### 3.2.3 Functional connectivity results

To explore whether interactional contexts modulate hemispheric balance in frontoparietal connectivity, we first conducted an exploratory data-driven analysis. Point-to-point scanning across all frontal-parietal electrode pairs was performed to identify electrode clusters showing the most robust context-dependent differences. This exploratory analysis identified four electrode clusters—left frontal (F3, FC3, FC1), left parietal (P3, P5, CP3), right frontal (F8, FT8), and right parietal (P4, CP6). A 2 (hemisphere) × 4 (context) repeated-measures ANOVA using these ROIs revealed a significant hemisphere × context interaction, F(3, 102) = 6.405, p <0.001, η^2^p = 0.049. Simple effect analysis indicated that the bilingual context was associated with reduced right-hemispheric and enhanced left-hemispheric connectivity relative to the English monolingual context in the exploratory analysis (ps <0.05) (Figure 10).

**Figure 10:**
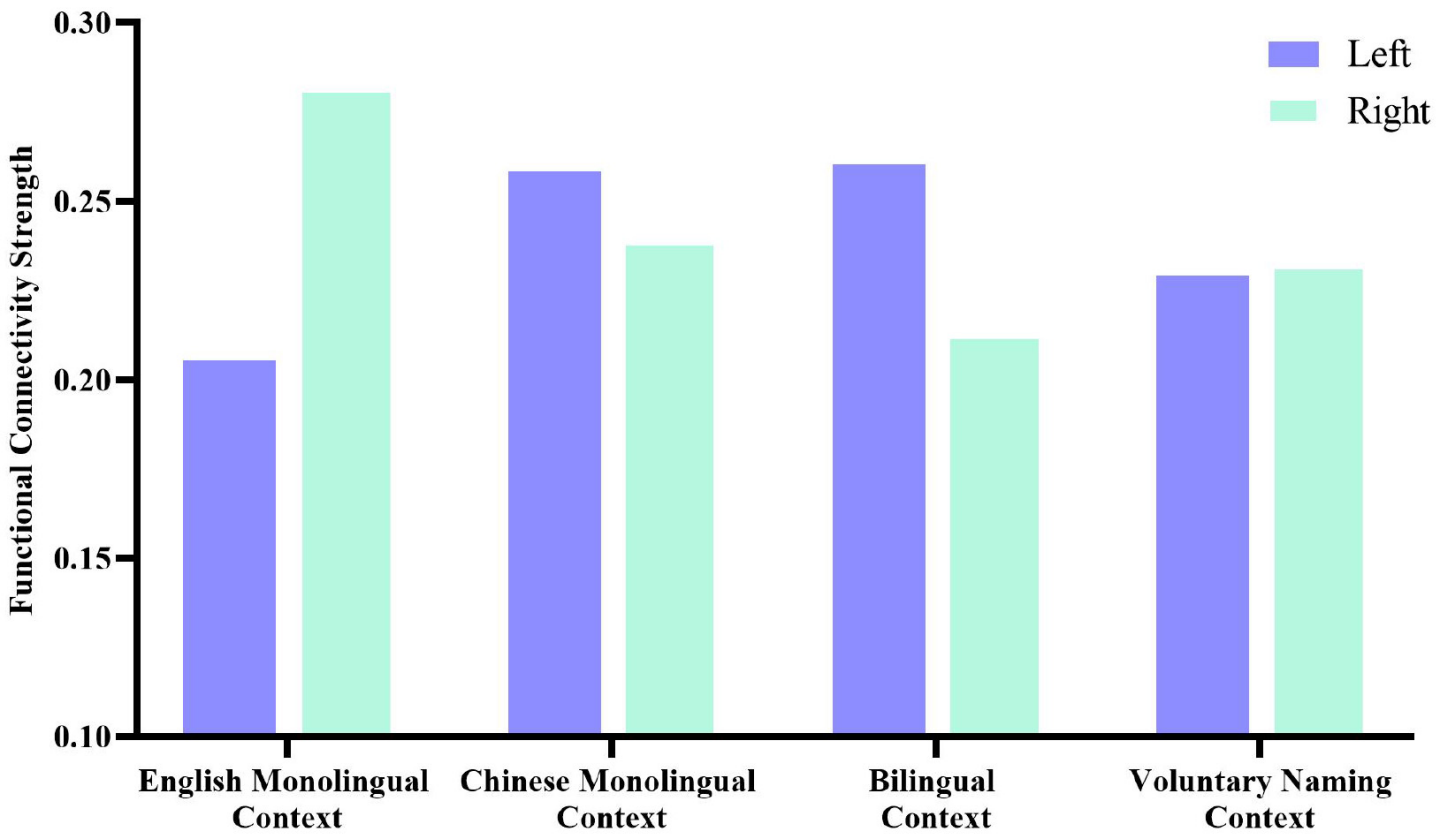
Functional connectivity strength of the frontoparietal network across the four language contexts for the left and right hemispheres.

Figure 11 presents the functional connectivity matrices for the four language contexts (English monolingual, Chinese monolingual, bilingual, and voluntary naming) within the 300–600 ms time window. The matrices display connectivity strength between left frontal (FC3, F3, FC1) and left parietal (CP3, P3, P5) regions, as well as between right frontal (F8, FT8) and right parietal (P4, CP6) regions. Warmer colors indicate stronger frontoparietal synchronization, while cooler colors indicate weaker synchronization. The matrices reveal that the bilingual context is associated with reduced connectivity in the right hemisphere and enhanced connectivity in the left hemisphere compared to the monolingual and voluntary naming contexts.

**Figure 11:**
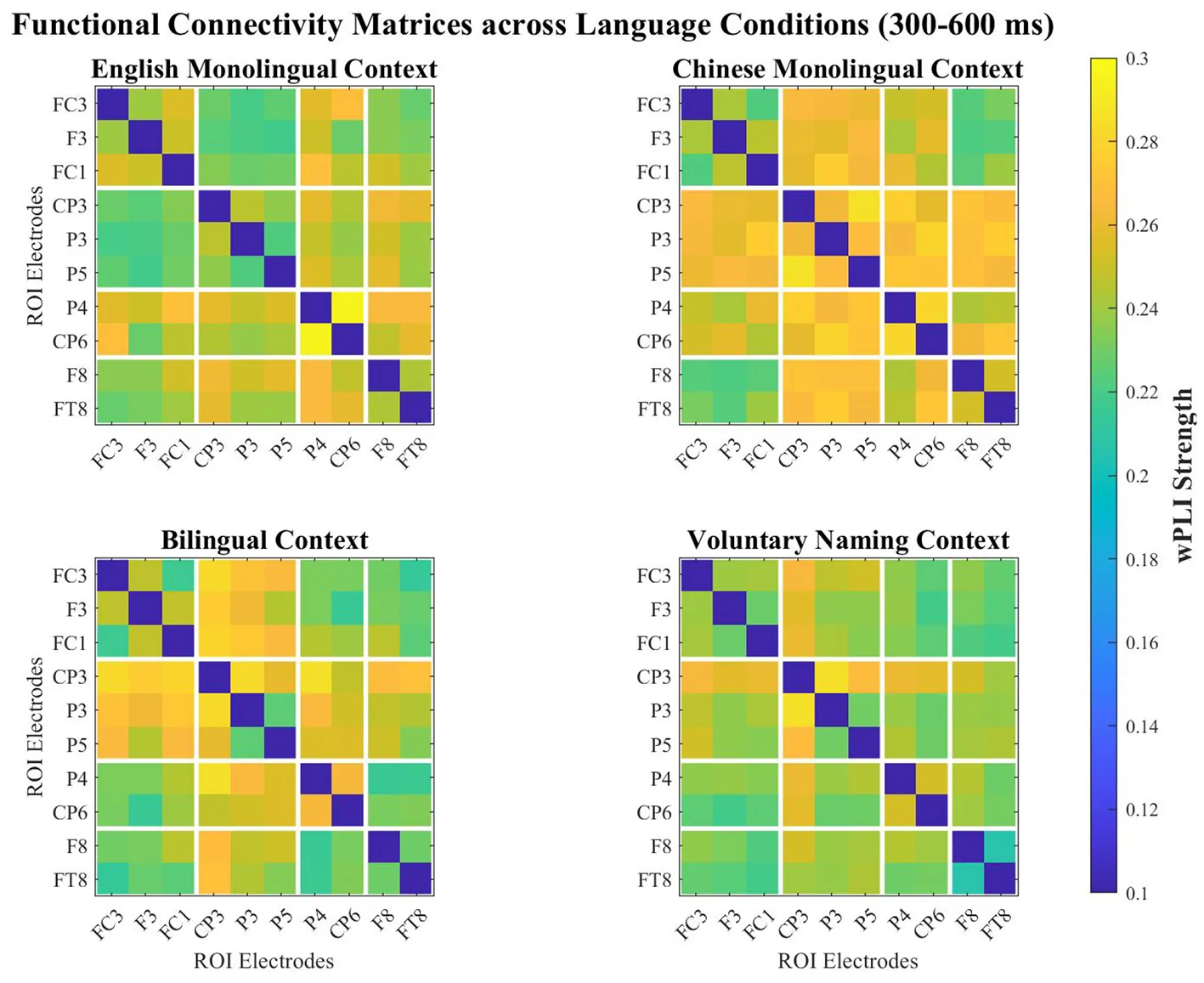
Functional Connectivity Matrices across Language Conditions (300–600 ms).

A one-way repeated measures ANOVA on right frontal-right parietal functional connectivity strength revealed a marginally significant main effect of interactional context [*F*(3, 102) = 2.638, *p* = 0.054, η^2^p = 0.072]. Pairwise comparisons revealed that connectivity strength in the bilingual context (*M* = 0.212, *SD* = 0.115) was significantly lower than in the English monolingual context (*M* = 0.281, *SD* = 0.165) (*p* = 0.046, Cohen's *d* = 0.543) (Figure 12).

**Figure 12:**
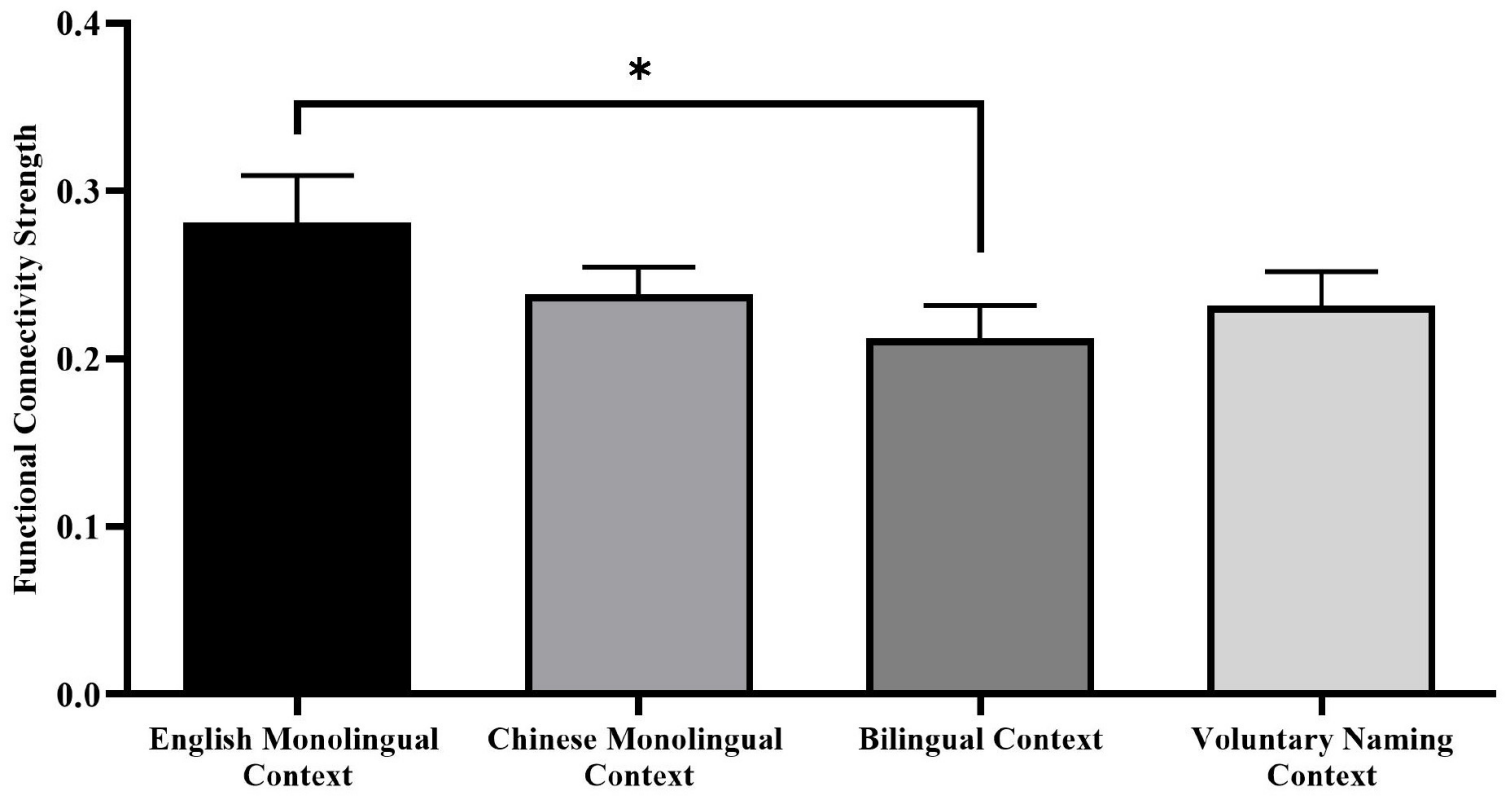
Right frontoparietal functional connectivity across language contexts. ^*^p < 0.05; ^**^p < 0.01; ^***^p < 0.001.

A one-way repeated measures ANOVA on left frontal-left parietal functional connectivity strength revealed a marginally significant main effect of interactional context [*F*(3, 102) = 2.517, *p* = 0.062, η^2^p = 0.069]. A paired-sample *t*-test comparing the English monolingual and bilingual contexts revealed that functional connectivity strength in the bilingual context (*M* = 0.260, *SD* = 0.113) was significantly stronger than in the English monolingual context (*M* = 0.206, *SD* = 0.098) (*t* = 2.510, *p* = 0.017, Cohen's *d* = 0.424) (Figure 13).

**Figure 13:**
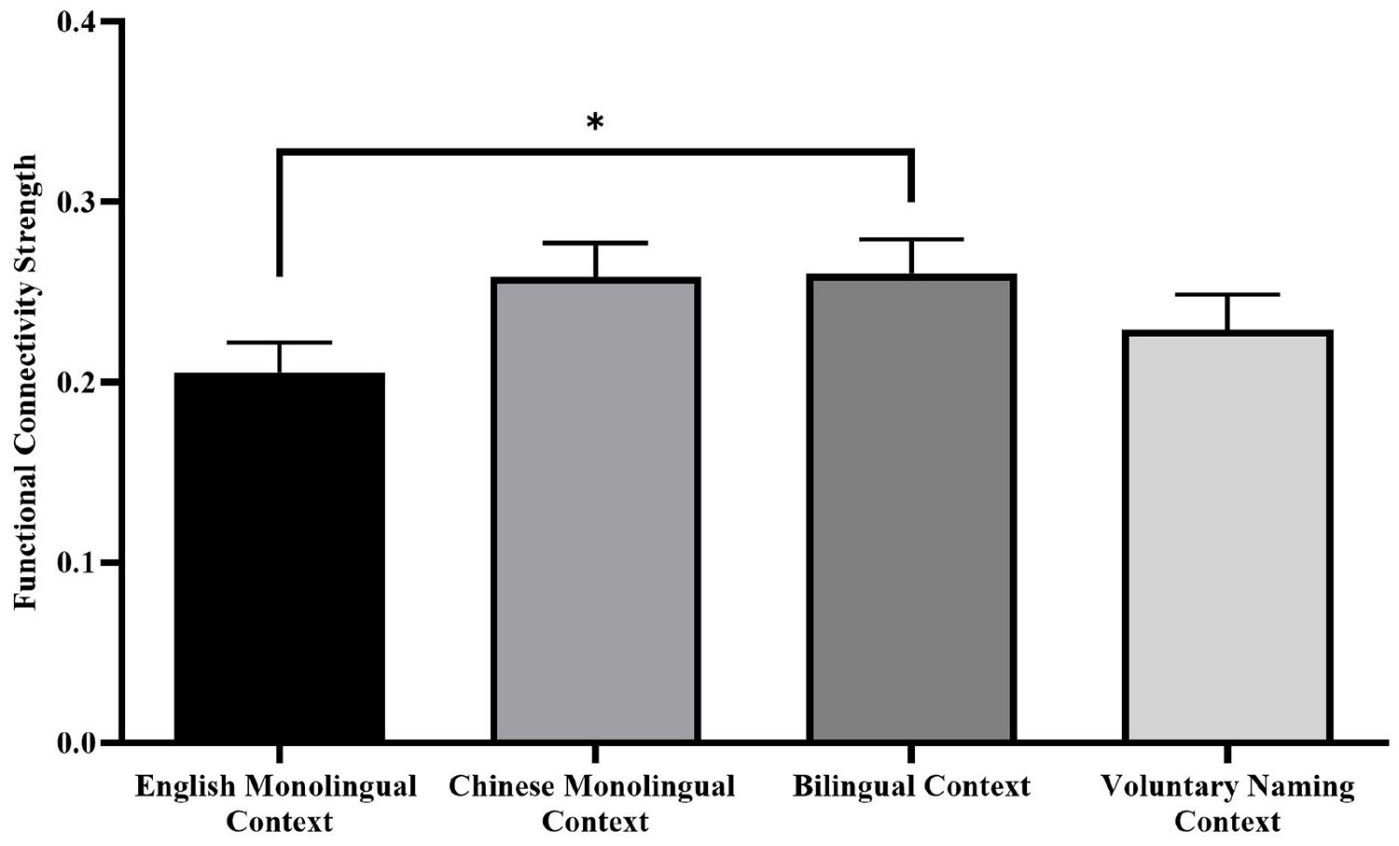
Left frontoparietal functional connectivity across language contexts. ^*^p < 0.05; ^**^p < 0.01; ^***^p < 0.001.

To assess the robustness of this exploratory finding and to address potential electrode-number confounds, we repeated the analysis using strictly homologous electrode clusters based on the left-hemisphere ROIs identified in the exploratory analysis (left: FC3, F3, FC1, CP3, P3, P5; right: F4, FC2, FC4, CP4, P4, P6). This symmetrical analysis showed a significant hemisphere × context interaction, *F*(3, 102) = 3.272, *p* = 0.024, η^2^p = 0.088. Simple main effect analysis showed that the left-right difference was significant only in the Chinese monolingual context, *F*(1, 34) = 7.327, *p* = 0.011, with no significant hemisphere differences in the bilingual context or other conditions (all ps > 0.05).

## 4 Discussion

The present study investigated how different interactional contexts dynamically modulate attentional control in bilingual speakers, guided by three hypotheses derived from an integrated theoretical framework. The findings provide a multi-level account of how bilingual contextual priming may facilitate attentional control.

At the behavioral level, the bilingual and voluntary naming contexts shortened antisaccade reaction times, providing support for the view that dual-language activation may facilitate subsequent attentional performance (Hypothesis 1). At the oscillatory level, reduced theta and delta power following these contexts suggested reduced control demands and enhanced neural efficiency, consistent with the prediction that contextual priming may trigger attentional resource reorganization (Hypothesis 2). At the network level, exploratory connectivity analysis using homologous electrode clusters revealed a significant hemisphere-by-context interaction, with left-right differences emerging specifically in the Chinese monolingual context. Notably, the bilingual-context effect observed in the initial data-driven exploration did not survive homologous ROI validation (Hypothesis 3). The N2 component, by contrast, showed no significant modulation across contexts, suggesting that the facilitatory effects may not be primarily driven by changes in early conflict monitoring.

Critically, the null N2 effect, the reduced oscillatory power, and the exploratory connectivity findings are not contradictory but complementary: they suggest that the effects of bilingual contextual priming may be more readily observable at the level of sensor-level connectivity patterns and sustained oscillatory dynamics, rather than transient ERP responses. These findings together support an integrated account in which bilingual contextual priming shifts participants toward the bilingual mode on Grosjean's (2023) language mode continuum, generating sufficient cognitive load to exceed the attentional resource reorganization threshold (Young and Stanton, 2002), thereby triggering a coordinated cascade of adaptation spanning neural networks, oscillatory dynamics, and behavioral performance.

### 4.1 Behavioral evidence for differential demands on attentional control across interactional context

The behavioral results clearly demonstrated that reaction times on the antisaccade task were significantly faster following the bilingual and voluntary naming contexts compared to the two monolingual contexts. These findings are consistent with the facilitatory effects of bilingual and voluntary naming contexts on attentional control: activation of the bilingual context optimized performance on subsequent non-linguistic attentional control tasks.

According to the malleable attentional resources theory (Young and Stanton, 2002), attentional resources are not a static, limited pool but rather a processing system that can be dynamically regulated according to task demands. When cognitive load remains below an individual's current regulatory capacity, resources are passively consumed; only when cumulative load exceeds a critical threshold does the system initiate resource reorganization and expansion, forming a more efficient control mode. In the present study, the significantly shortened reaction times in the bilingual and voluntary naming contexts may be attributed to the high language control demands imposed by these contexts, which generated substantial cognitive load (Green and Abutalebi, 2013). This load exceeded the regulatory threshold of the attentional system, thereby triggering resource reorganization (Li et al., 2026). This reorganized, more efficient control mode persisted, enabling subsequent antisaccade performance with reduced attentional resource investment, as reflected in significantly shortened reaction times. This indicates that the effect of interactional context operates through activating adaptive reorganization of the attentional system, thereby optimizing its overall operational mode (Bialystok, 2024).

The behavioral data show that any environment requiring simultaneous activation of two languages, whether the bilingual context or the voluntary naming context, produces significant facilitatory effects, suggesting that any context requiring dual-language activation may induce similar attentional system adaptation effects. This finding aligns with Grosjean's (2023) language mode theory, which predicts that the joint activation of both languages in bilingual mode imposes greater demands on selective attention than monolingual mode. Critically, the present results demonstrate that these contextual effects can emerge rapidly within a single experimental session, consistent with Grosjean's observation that movement along the language mode continuum can occur quickly in response to situational factors (Grosjean, 2023). Our findings align with and extend previous work on language context and cognitive control. Jiao et al. (2020a,b) demonstrated that a mixed-language production context modulated electrophysiological responses during a subsequent Flanker task, suggesting that language production contexts engage cognitive control processes that carry over to subsequent task performance.

### 4.2 Electrophysiological evidence for attentional control modulation by language contexts

We first examined the N2 component at fronto-central electrodes. A one-way repeated-measures ANOVA revealed no significant main effect of interactional context. This null finding is not unprecedented. Using an N-back task, Pereira Soares et al. also found no ERP correlates of bilingual experience, while significant effects emerged in time-frequency representations. This pattern of null effects in ERPs alongside significant effects in time-frequency measures mirrors the present findings and suggests that ERPs may be less sensitive for capturing within-bilingual individual differences (Pereira Soares et al., 2024). For the present study, this interpretation suggests that the null N2 effect does not indicate a failure of attentional adaptation, but rather that the N2 component may not be the most sensitive index for detecting the efficiency-related changes induced by contextual priming.

As outlined in Section 1.5, we hypothesized that oscillatory dynamics would provide a more sensitive index of enhanced neural efficiency than transient ERP responses, given their reflection of sustained resource allocation over longer time windows (Cavanagh and Frank, 2014; Harmony, 2013). Time-frequency analysis is advantageous because it can reveal non-phase-locked (induced) oscillations that are canceled out in traditional ERP averaging (Dickter and Kieffaber, 2014). These induced oscillations reflect cognitive processes—such as sustained resource allocation—that are not strictly time-locked to stimulus onset (Singhal et al., 2023). Consequently, time-frequency measures offer greater sensitivity to subtle, sustained adaptation effects that may arise from contextual priming, particularly in within-group designs (Pereira Soares et al., 2024). This methodological choice is consistent with recent work by Xie et al. (2024), who argued that time-frequency representations are generally better suited for capturing bilingual effects in such designs because they provide sufficient granularity to detect effects that might not emerge in ERPs alone (Xie et al., 2024).

Theta oscillations are well established markers of cognitive control and conflict monitoring, extensively documented to be closely associated with conflict detection and cognitive adjustment processes (Cavanagh and Frank, 2014). Delta oscillations are closely associated with processes involved in suppressing task-irrelevant information, with increased delta power reflecting neural activity underlying active interference suppression in tasks requiring such inhibition (Harmony, 2013). In the present study, significantly reduced theta and delta power were observed in the antisaccade task following bilingual and voluntary naming contexts. Several interpretations of this reduction are possible. One account is that reduced theta and delta power reflect reduced control demands due to more efficient processing (Cohen, 2011; Gratton et al., 2018). According to the malleable attentional resources theory (Young and Stanton, 2002), the bilingual priming context may have provided sufficient cognitive load to exceed the resource reorganization threshold during the naming phase, thereby reducing the need for conflict monitoring in the subsequent antisaccade task. An alternative interpretation is that lower oscillatory power reflects increased task automaticity or reduced stimulus unpredictability following priming (Cavanagh and Frank, 2014). These accounts are not mutually exclusive, and all converge on the conclusion that the bilingual and voluntary naming contexts reduced the control demands of the subsequent antisaccade task.

Critically, both the reduction in neural oscillatory power and the superior behavioral performance were observed following the same priming contexts. The co-occurrence of better performance and lower neural activity is consistent with the core signature of enhanced neural efficiency (Bialystok, 2024). Specifically, the “dual-high” pattern (high delta, high theta) observed following the English monolingual context may reflect a high-consumption processing mode: the system failed to effectively reduce control demands during the priming phase, resulting in sustained investment of monitoring resources during the subsequent task. In contrast, the “dual-low” pattern observed following the bilingual context reflects reduced control demands. This “better performance–lower neural activity” pattern is consistent with the broader principle of neural efficiency, which characterizes the adaptationist perspective (Bialystok, 2024). However, it is important to clarify that Bialystok's framework does not make specific predictions about neural oscillatory measures, and our findings should be interpreted as demonstrating a pattern that is conceptually consistent with rather than directly supportive of this perspective. Moreover, as discussed below, our priming paradigm examines immediate contextual effects, whereas the adaptationist framework concerns cumulative adaptations to sustained experience.

The present results reveal that brief exposure to bilingual or voluntary naming contexts can immediately enhance neural efficiency during subsequent executive control tasks. This enhancement is manifested both in reduced theta/delta power and in better behavioral performance. This online priming effect may reflect a transient form of attentional resource reorganization. Converging evidence from long-term language experience study further supports this interpretation. Using resting-state fMRI, Li et al. found that bilinguals with greater language diversity, measured by language entropy across social contexts, exhibited lower BOLD signal variability and higher brain network specialization, neural patterns associated with more efficient conflict monitoring and task-switching. Thus, both immediate contextual priming and sustained diverse language use appear to promote attentional system efficiency through potentially overlapping neural mechanisms, bridging online adaptation and long-term neuroplasticity (Li et al., 2021).

### 4.3 Exploratory functional connectivity analysis

The functional connectivity analysis was treated as exploratory, given the sensor-level nature of EEG connectivity and the absence of prior ROI hypotheses. An initial data-driven exploration suggested that the bilingual context was associated with reduced right-hemispheric and enhanced left-hemispheric connectivity. However, when the analysis was repeated using strictly homologous electrode clusters to address potential electrode-number confounds, this bilingual-context effect did not survive. Instead, the homologous ROI analysis revealed a significant hemisphere × context interaction, with left-right differences emerging specifically in the Chinese monolingual context.

The discrepancy between the data-driven and homologous ROI findings highlights the sensitivity of sensor-level connectivity measures to ROI definition. While the exploratory analysis provided initial clues about potential context-dependent connectivity changes, the lack of robustness under more rigorous methodological control suggests that these effects should not be overinterpreted. This pattern is consistent with the view that sensor-level EEG connectivity measures are inherently limited in their ability to index cortical network reorganization, as volume conduction and field spread can mix signals from multiple neural sources at the sensor level (Bastos and Schoffelen, 2016). Moreover, ROI extraction methods significantly influence connectivity estimates derived from sensor-level data (Brkić et al., 2023), and source-level analyses are required to draw valid inferences about connectivity between specific cortical regions (Schoffelen and Gross, 2009).

The finding that the bilingual context produced robust behavioral and oscillatory effects without corresponding robust connectivity changes may indicate that the neural mechanisms underlying bilingual facilitation operate at different levels of neural organization. Behavioral and oscillatory measures may index global resource allocation and neural efficiency, whereas hemispheric balance in frontoparietal connectivity may be more sensitive to ROI definition than to the broader interactional context.

Critically, these connectivity findings should be interpreted with caution. Sensor-level analyses do not, by themselves, permit direct inference about connectivity between specific cortical regions. Therefore, these findings are considered hypothesis-generating rather than conclusive. Future research employing source-localized EEG or fMRI is needed to determine whether the observed patterns reflect genuine cortical network asymmetries.

## 5 Limitations

Several methodological limitations should be acknowledged when interpreting the present findings. First, our behavioral task did not include eye-tracking, which prevented us from confirming that participants executed saccades as instructed and from distinguishing between correct antisaccades and prosaccades that were subsequently corrected. Consequently, our reaction time measure reflects a composite of cognitive and motor processes rather than a pure index of inhibitory control (Hutton, 2008). Additionally, the absence of a prosaccade block or interleaved prosaccade trials limits our ability to determine whether the observed contextual effects reflect inhibition specifically or broader factors such as attention allocation, perceptual processing, or motor responding. Furthermore, our functional connectivity analysis was based on scalp-level electrode ROIs and did not include source localization. The results were ROI-dependent. This sensitivity underscores the exploratory nature of sensor-level connectivity measures and highlights the need for validation through source-localized EEG or fMRI.

Second, our sample size was modest, particularly after exclusions (final *n* = 35). A *post hoc* power analysis using G^*^Power indicated that, assuming a small effect size (*f* = 0.10) consistent with meta-analytic estimates of bilingual effects (Donnelly et al., 2019; Lehtonen et al., 2018), a substantially larger sample would be required to achieve 80% power. Although our within-subjects design and focus on neural measures partially mitigate this limitation, the present findings should be considered preliminary and in need of replication with larger samples.

Third, the present study examined only immediate effects of contextual priming on subsequent task performance. The duration of these facilitatory effects remains unknown, and whether such transient modulation can accumulate into longer-term cognitive advantages through repeated exposure requires investigation through delayed post-tests or longitudinal designs. Relatedly, the graded effects of long-term bilingual experience, such as the continuous influence of age of acquisition and proficiency on cognitive control, were not systematically tested here, as our primary focus was on immediate contextual modulation. Future studies combining immediate priming manipulations with comprehensive measures of long-term language experience are needed to bridge these two timescales of bilingual adaptation.

## 6 Conclusion

The present study provides evidence that priming by bilingual and voluntary naming contexts may facilitate attentional control in bilingual speakers. This facilitation is suggested by behavioral improvements (shortened reaction times) and neural changes (reduced delta/theta power), which are consistent with enhanced efficiency, as well as preliminary, ROI-dependent connectivity patterns that require validation by source-localized methods.

## Data Availability

The datasets presented in this study can be found at the Baidu Netdisk repository. These data can be accessed at https://pan.baidu.com/s/18W24JUZUuZt-fb4x_2Tp5A, with the access code 7070.
